# Lighting system bioinspired by *Haworthia obtusa*

**DOI:** 10.1038/s41598-020-68196-8

**Published:** 2020-07-09

**Authors:** Hiroki Gonome, Kazuya Watanabe, Kae Nakamura, Takahiro Kono, Jun Yamada

**Affiliations:** 1 Department of Mechanical Systems Engineering, Yamagata University, 4-3-16 Jonan, Yonezawa, Yamagata 992-8510 Japan; 2 Department of Mechanical Engineering, Shibaura Institute of Technology, 3-7-5 Toyosu, Koto-ku, Tokyo, 135-8548 Japan; 3 Department of Precision Machinery Engineering, Nihon University, 7-24-1 Narashinodai, Funabashi, Chiba 274-8501 Japan

**Keywords:** Nanoparticles, Optical techniques, Plant sciences, Mechanical engineering

## Abstract

Electricity plays an important role in modern societies, with lighting and illumination accounting for approximately one-fifth of the global demand for electricity. *Haworthia obtusa* has the remarkable ability to collect solar light through a so-called ‘window’ which allows it to photosynthesise in the dark. Inspired by this unique characteristic, we developed a novel lighting system that does not use electricity. The ‘window’ of *H. obtusa* is replicated using a scattering medium that collects solar light and guides it to an optical fibre. The optical fibre then carries the light indoors, where illumination is needed. The efficacy of this unique lighting system was confirmed both numerically and experimentally. The developed system should help in lowering energy consumption.

## Introduction

Electricity is used extensively in modern societies, and devising new methods for saving electrical energy is a perennial goal in engineering. Lighting and illumination account for virtually one-fifth of the global demand for electricity. Thus, there is a strong need to develop lighting systems that do not use electricity.

**Figure 1:**
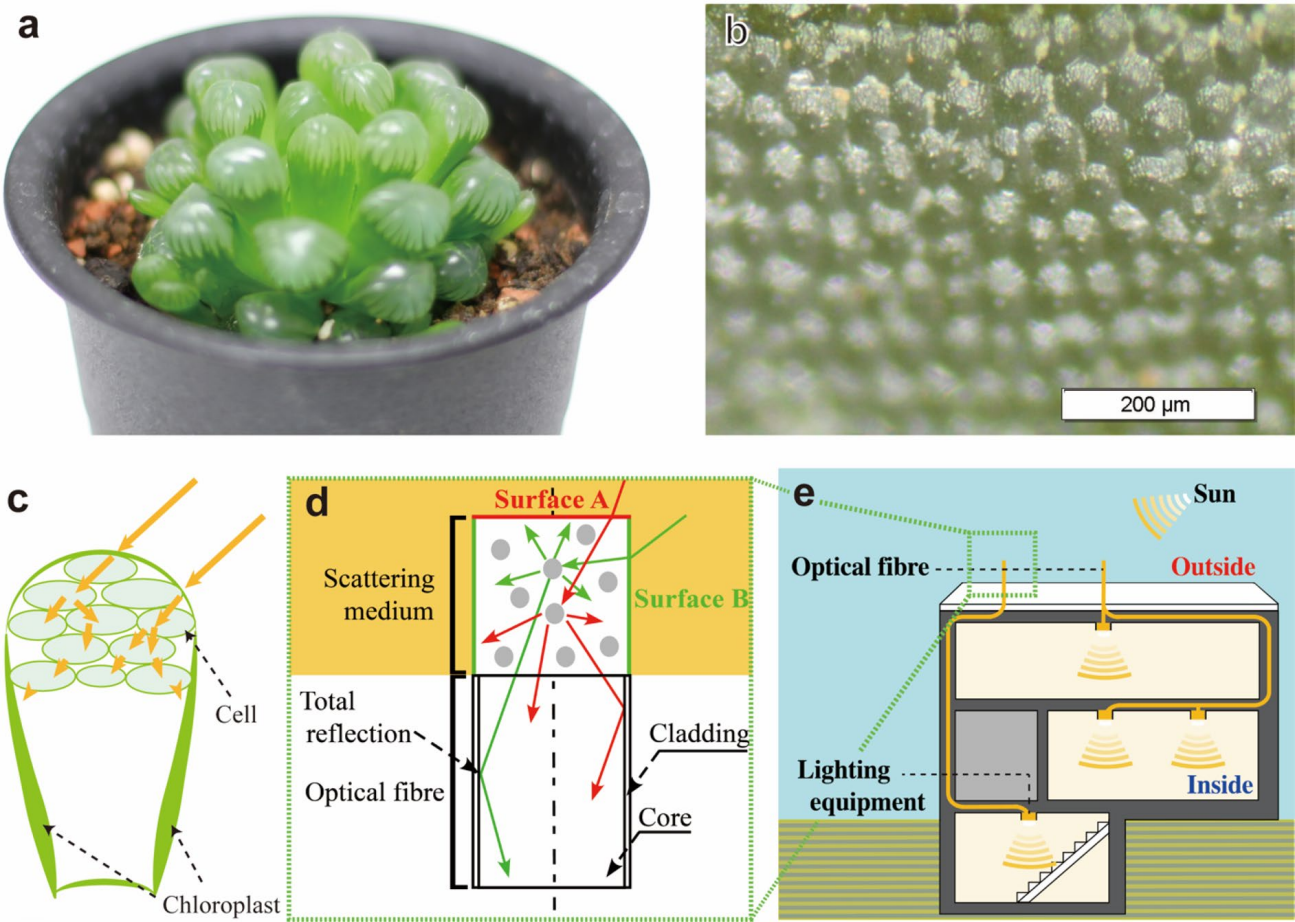
Concept of lighting system bioinspired by *Haworthia obtusa*. (**a**) Photograph of *H. obtusa*. (**b**) Optical microscopy image of the inside of *H. obtusa*. (**c**) Schematic of mechanism for collecting and guiding solar light to chloroplasts through transparent cells in *H. obtusa*. (**d**) Schematic of proposed optical fibre system with low-cost scattering medium attached at edge. (**e**) Schematic showing use of proposed optical fibre for indoor lighting.

Plants also need light for photosynthesis, and many have evolved to be able to photosynthesise^1^ and survive in inhospitable environments. For example, *Haworthia obtusa* exhibits a remarkable ‘window’ for collecting solar light. This unique body structure enables *H. obtusa* to photosynthesise in the dark. In this ‘window’, light is transmitted through transparent cells, scattered by the cell walls, and eventually guided to the chloroplasts (Fig. 1).

The unique characteristics exhibited by various organisms in nature have long inspired scientists to develop optical materials and devices that mimic these functionalities^2–11^. In this study, we developed a novel lighting system which directly uses sunlight by mimicking the characteristics of *H. obtusa* and can be a substitute for conventional lighting systems, which require electricity. In the developed system, a scattering medium is attached at the edge of an optical fibre to expand the receivable angle of the optical fibre, like the ‘window’ of *H. obtusa*. Optical fibres consist of a central core and the surrounding cladding and transfer light by total reflection at a narrow acceptance angle. Usually, only the top surface of optical fibres can receive light. In the developed system, however, the receivable surface is expanded to include not only the top surface (surface A) but also the side surface (surface B); this is accomplished by attaching the scattering medium to the fibre. Therefore, the transferred light flux increases because the previously nonreceivable light is guided at an acceptable angle. The captured light is thus carried indoors by the optical fibre, where illumination is needed.

Several researchers^12,13^ have proposed new lighting technologies for buildings that utilise sunlight and have assessed their performances for the benefit of those in the building industry. Wittkopf et al.^14^ reported a novel method for studying how well nonimaging daylight collectors pipe diffuse daylight into long horizontal funnels for illuminating deep buildings. However, this method is too expensive for personal use as well as difficult to implement^15^. As a substitute for expensive tube-like light guidance systems, optical fibres have attracted a lot of attention. Xue et al.^16^ developed a novel sunlight concentrating and guiding system for optical fibres for daylighting applications. They constructed a sunlight concentrator unit that includes a novel mirror image co-focus compound parabolic concentrator. Sapia^17^ and Han et al.^18^ used an optical fibre and a solar concentrator to fabricate a daylighting system for buildings. In this system, a sun-tracking parabolic concentrator collects sunlight. Generally, optical fibres require a concentrator to collect an adequate amount of sunlight, as their acceptance angle is small. However, concentrators are also too expensive for home use. Thus, any lighting system that works without electricity must also be low-cost.

In this study, a novel lighting system that does not require an expensive sunlight concentrator was developed. The system is based on a low-cost scattering medium, which is attached at the edge of an optical fibre to increase its receivable angle. To evaluate the performance of the lighting system, radiative transfer in the optical fibre was simulated using the Monte Carlo method. Moreover, the optical fibre was also evaluated experimentally. Finally, the experimental and simulation results were compared with the aim of improving the performance of the lighting system.

## Results

### Numerical modelling

**Figure 2:**
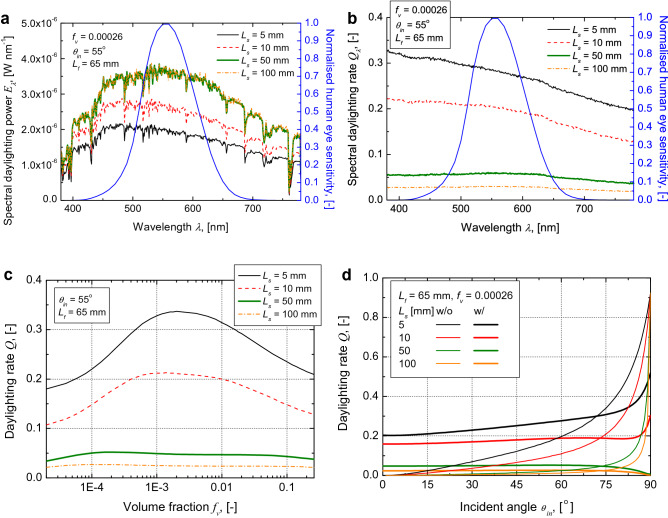
Effects of length, volume fraction, and incident angle of scattering medium consisting of Al_2_O_3_ particles on daylighting rate. (**a**) Calculated spectral daylighting rate when *f*_*v*_ is 0.00026, incident angle is 55°, length of optical fibre is 65 mm, and length of scattering medium is 5 mm (black curve), 10 mm (red dash curve), 50 mm (bold green curve), and 100 mm (orange chain curve). Normalised human eye sensitivity is also shown (blue dot curve). (**b**) Calculated spectral daylighting power when *f*_*v*_ is 0.00026, incident angle is 55°, length of optical fibre is 65 mm, and length of scattering medium is 5 mm (black curve), 10 mm (red dash curve), 50 mm (bold green curve), and 100 mm (orange chain curve). Normalised human eye sensitivity is also shown (blue dot curve). (**c**) Effect of volume fraction on daylighting rate when incident angle is 55°, length of optical fibre is 65 mm, and length of scattering medium is 5 mm (black curve), 10 mm (red dash curve), 50 mm (bold green curve), and 100 mm (orange dot curve). (**d**) Effect of incident angle on daylighting rate when *f*_*v*_ is 0.00026, length of optical fibre is 65 mm. The cases of scattering medium without (w/o) particle (normal curve) and with (w/) particle (bold curve) were calculated and the length of the scattering medium was 5 mm (black curve), 10 mm (red curve), 50 mm (green curve), and 100 mm (orange curve).

We simulated radiative transfer in the optical fibre with the scattering medium. Al_2_O_3_ particles with a diameter of 0.7 μm were used as the scattering particles. Al_2_O_3_ does not exhibit spectral dependence on the solar spectrum. Therefore, its spectral daylighting rate is almost constant in this region (Fig. 2a). The daylighting rate decreases with an increase in the length of the scattering medium, because the daylighting power decreases along the length. However, the total spectral daylighting power increases with an increase in the length of the scattering medium, because the incident flux increases with the irradiated area (Fig. 2b). In addition, the daylighting power from the side surface is much higher than that from the top surface.

The volume fraction also affects the daylighting rate (Fig. 2c). The daylighting rate increases with the volume fraction of the scattering medium initially but then decreases once the degree of scattering becomes high, causing the amount of power scattered or reflected to increase. The scattering coefficient of the particle cloud increases with its volume fraction. These factors suggest that it is essential to control the scattering coefficient of the scattering medium (i.e., the volume fraction of the scattering medium) in order to maximise the performance of the proposed system.

In reality, the incident angle of solar light changes with the season and time of day. However, the daylighting rate of the optical fibre with the scattering medium with particles remains virtually constant with changes in the incident angle, in contrast to the case for optical fibre with the scattering medium without particles (Fig. 2d). This highlights the usefulness of the bioinspired fibre system as a lighting source. The daylighting rate increases with the incident angle when *L*_*s*_ = 5 and 10 mm because these lengths correspond to the critical angle of the optical fibre. On the other hand, the daylighting rate decreases with the incident angle when *L*_*s*_ = 50 and 100 mm because, in these cases, the daylighting power from the top surface decreases.

### Experimental evaluation

**Figure 3:**
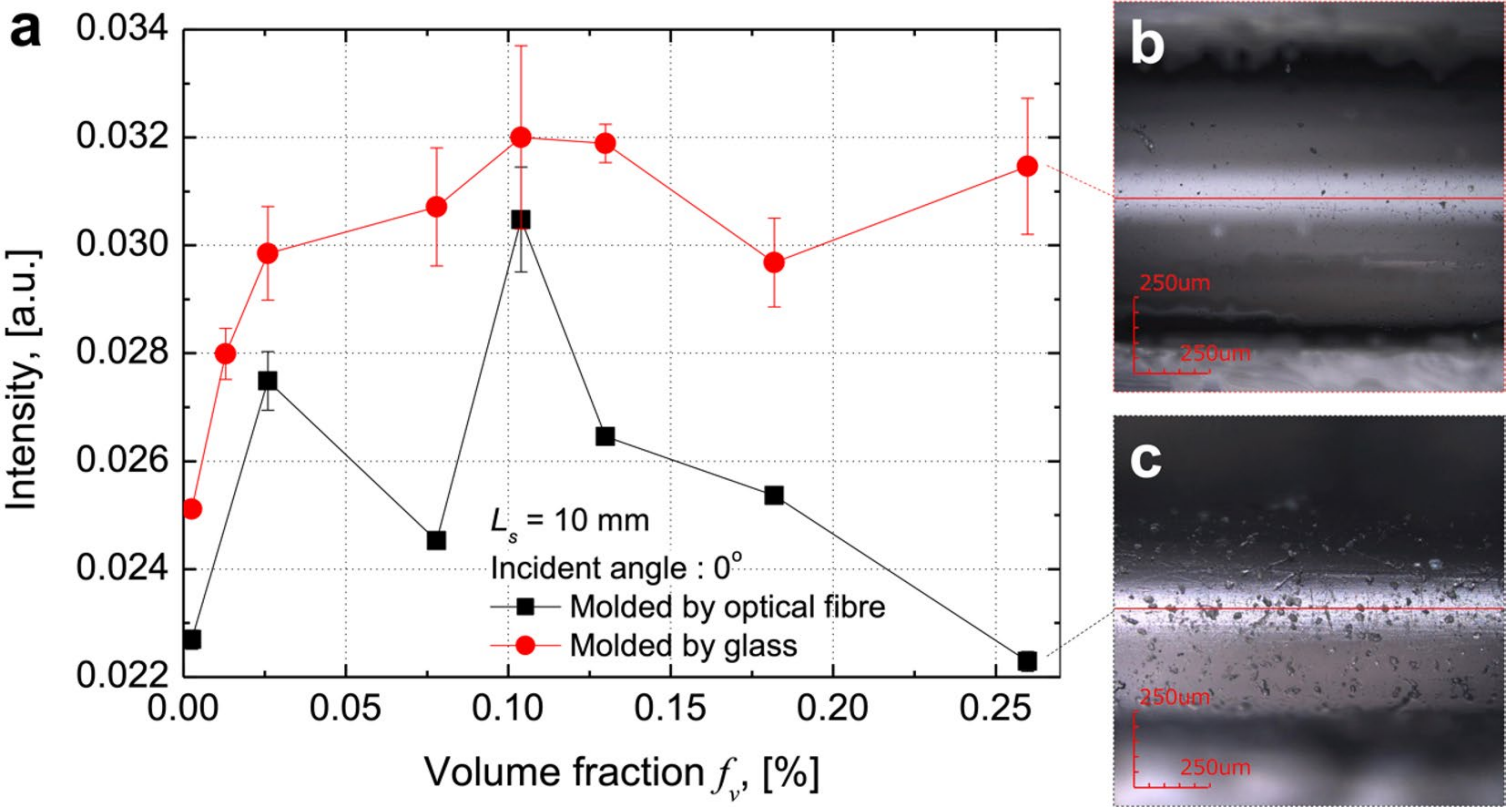
Experimental evaluation of performance of proposed lighting system. (**a**) Measured illumination power when length of scattering medium is 10 mm and incident angle is 0° for optical fibre moulded using optical fibre (black square curve) and glass (red circle curve). (**b**) Laser microscopy image of surface of optical fibre moulded using glass. (**c**) Laser microscopy image of surface of optical fibre moulded using optical fibre.

We evaluated the lighting performance of the proposed optical fibre system experimentally. It was found that, during the fabrication of the proposed system, the surface roughness plays a crucial role. The light intensity of an optical fibre moulded using glass was higher than that of a fibre moulded using an optical fibre (Fig. 3a). Thus, moulding with an optical fibre resulted in a rougher surface (Fig. 3b) as compared with moulding with glass (Fig. 3c). Rough surfaces promote the diffuse reflection of light at the boundary between the scattering medium and air because the amount of radiation entering the scattering medium decreases. The simulations showed that the light intensity was the highest when the volume fraction of the scattering medium was approximately 0.001 (Fig. 2c); this was true for both samples.

### Comparison of experimental and simulation results

To optimise the proposed lighting system in order to maximise its performance, we compared the experimental and simulation results. The light intensity for a volume fraction of 0.00026 was used as the benchmark, and the increase rate, *R*, was calculated as follows:

$$R=\frac{Q_{{f}_{v}}}{Q_{{f}_{v}\,=\,0.00026}}.$$

**Figure 4:**
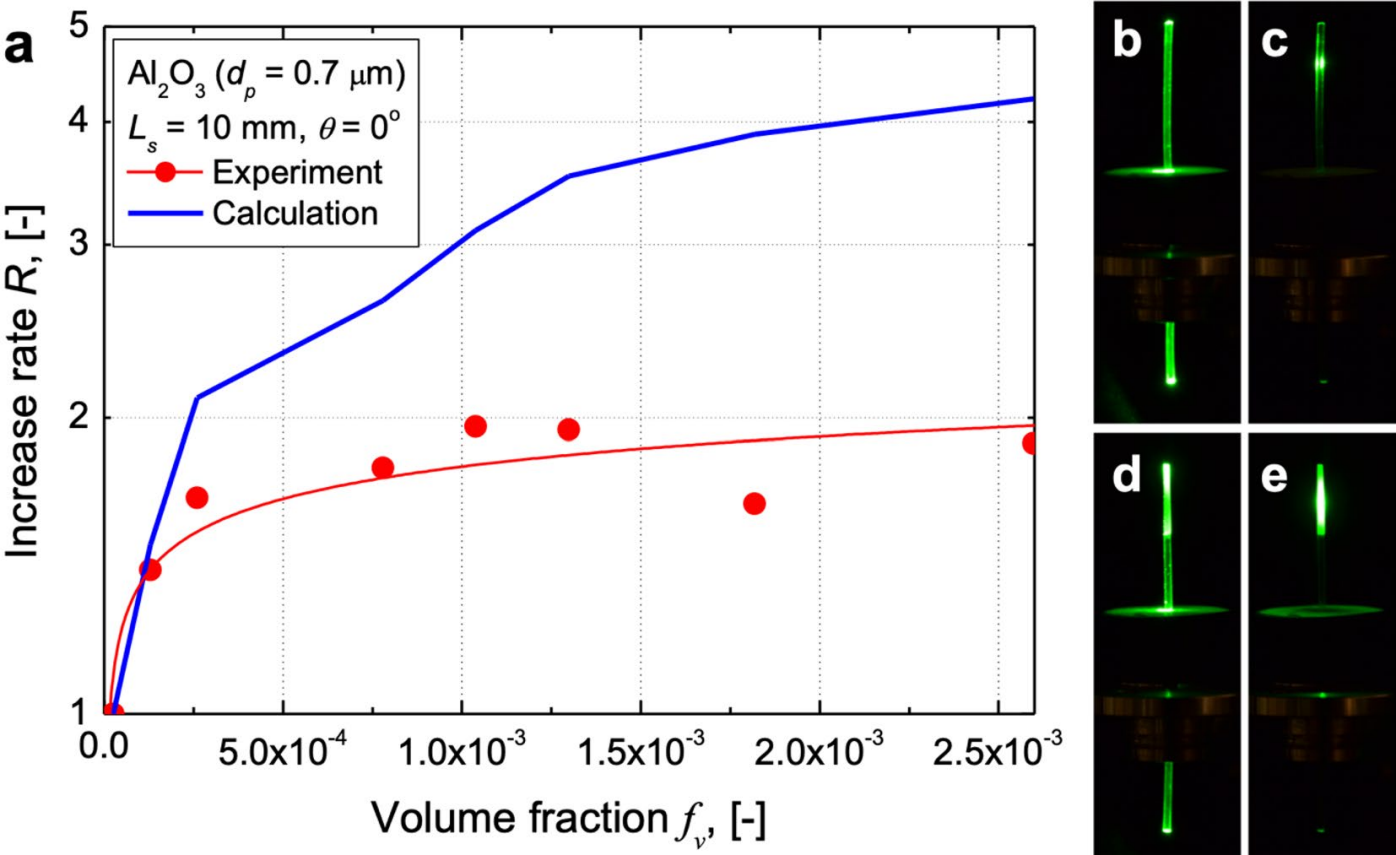
Comparison of experimental and simulation results. (**a**) Increase in illumination power as function of volume fraction of scattering medium when length of scattering medium is 10 mm and incident angle is 0° as determined experimentally (red circle curve) and through calculations (blue bold curve). (**b**) Visualisation of light propagation in raw optical fibre when incident angle is 90°. (**c**) Visualisation of light propagation in raw optical fibre when incident angle is 0°. (**d**) Visualisation of light propagation in bioinspired optical fibre when incident angle is 90°. (**e**) Visualisation of light propagation in bioinspired optical fibre when incident angle is 0°.

The experimentally determined and simulated values of *R* showed similar dependences on the volume fraction and increased with it (Fig. 4a). However, the experimental *R* values of the sample moulded using glass were smaller than the calculated ones. This was because of the imperfections in the adhesive layer between the optical fibre and the attached scattering medium. The layer of adhesive used during sample fabrication caused light to be reflected at the boundary, thus decreasing the amount of light that propagated from the scattering medium to the optical fibre (Fig. 4b–e).

## Discussion

Inspired by *H. obtusa*, we developed a new lighting system that does not use electricity. It collects solar light through a scattering medium. We determined the radiative transfer characteristics of the scattering medium in order to evaluate the performance of the lighting system. For optimal lighting, the volume fraction of the scattering medium and the set angle are important for controlling the scattering coefficient and incident angle. Strong scattering has an adverse effect on the lighting performance. Hence, it is essential to determine the optimal scattering coefficient.

During the numerical and experimental evaluations, the maximum light intensity for a single optical fibre was low. However, a bundle of fibres would be able to transfer more light. Moreover, the lighting performance of individual optical fibres can be improved. The scattering medium was fabricated using spherical Al_2_O_3_ particle and a fibre resin. Therefore, its scattering phase function assumed to be isotropic. To improve the lighting performance, the directional control of light is important and may be realised using nanorods or nanofibres because of their anisotropic scattering properties.

The surface roughness of the optical fibre and the boundary between the scattering medium and the fibre also have a determining effect on the lighting performance. These factors can be optimised during the manufacturing process. While the performance of the proposed optical fibre system in the laboratory was limited, we believe that the system can be improved such that it is suitable for practical use. The scattering medium can collect significantly more light than the raw optical fibre (Fig. 4b–e). Thus, this system will help reduce energy consumption. Moreover, based on this study, we expect that more bioinspired optical technologies will be developed in the future.

## Methods

### Radiative transfer analysis

To estimate the performance of the lighting system, radiative transfer in the optical fibre with the scattering medium was simulated using the Monte Carlo method^19,20^. The radiative transfer in the optical fibre can be expressed by the following equation:

$$\frac{d{I}_{\lambda }\left(s,\Omega \right)}{ds}=-{\left({\sigma }_{s}+\alpha \right)I}_{\lambda }\left(s,\Omega \right)+\frac{{\sigma }_{s}}{4\pi }{\int }_{4\pi }{I}_{\lambda }\left(s,\Omega \right)\Phi \left({\Omega }^{^{\prime}}\to\Omega \right)d\Omega ,$$

where *I* is the intensity of the incident radiation, *s* is the path length, σ_*s*_ is the scattering coefficient of the medium, *α* is its absorption coefficient, and Φ is the scattering phase function. Al_2_O_3_ (refractive index *n* = 1.77) particles with a diameter, *d*_*p*_, of 0.7 μm was used as the scattering particles. The radiative properties (e.g., absorption and scattering coefficients) of the scattering particles were calculated based on the Mie scattering theory^21^. During the numerical simulations (See Supplementary Fig. S1), the diameter of the optical fibre was taken to be 1 mm. The surrounding medium was air with *n* = 1.00. The refractive indexes of the core, *n*_1_, and scattering medium, *n*_*s*_, were set to 1.49, while that of the cladding, *n*_2_, was set to 1.40. The scattering coefficient of the pellucid part was 0 mm^-1^. Further, based on ASTM G197-08, it was assumed that the incident flux, *ϕ*_in_, irradiates at an angle of *θ*_*in*_. The length of the scattering part, *L*_*s*_, was kept variable. Finally, the length of the optical fibre, *L*_*f*_, was set at 65 mm. As stated above, the scattering phase function was assumed to be isotropic, and the scattering medium was assumed to be nonabsorbing (*α* = 0 mm^-1^). The number of photons incident at the top surface was 10^6^, while the number incident at the side surface was 90,000 × *L*. The incident photons were scattered in the scattering part and transferred to the pellucid part. The daylighting rate, *Q*, can be expressed as

$$Q=\frac{{\int }_{380}^{780}{E}_{\lambda }d\lambda }{{\int }_{380}^{780}{\phi }_{in, \lambda }d\lambda \times A},$$

where *E*_*λ*_ is the spectral daylighting power and *A* is the irradiated area.

### Sample preparation

As stated previously, we fabricated the lighting system by attaching a scattering medium at the edge of an optical fibre. The bioinspired optical fibre was produced by joining an optical fibre (CK-40, Mitsubishi Chemical Corporation) and a narrow glass tube; their diameter was 1 mm and length was 20 mm (See Supplementary Fig. S2a). The mould itself was made of a silicon resin (KE-1603, Shin-Etsu Chemical). The scattering medium was made of an acrylic resin (SS101, Epoch) and Al_2_O_3_ particles (AKP-3000, Sumitomo Chemical Co. Ltd.) with a diameter, *d*_*p*_, of 0.7 μm. The weight of the resin matrix and scattering particles was measured with an electronic balance (XFR-225 W, Shinko Denshi). The volume fraction of the particles in the scattering medium *f*_*v*_ can be evaluated using the following equation:

$${f}_{v}=\frac{\left({x}_{p}/\rho_{x}\right)}{\left({x}_{p}/\rho_{x}\right)+{V}_{y}},$$

where *x*_*p*_ is the weight of the scattering particles, *ρ*_*x*_ is the density of the scattering particles, and *V*_*y*_ is the volume of the resin matrix. To prevent the agglomeration of the scattering particles, an ultrasonic device (ASU-20, ASONE) was used for 60 min. The resin and particles were mixed using a super mixer (AR-100, Thinky); the mixing time was set at 2 min for stirring and 1 min for defoaming. A mould release agent (GA-7500, Daikin Industries) was sprayed on the mould. Subsequently, the mixture was injected into the mould and allowed to stand for one day. The solidified scattering medium was then removed from the mould. The scattering medium and optical fibre were joined together using an acrylic adhesive (AS001-02, ACRYSUNDAY Co.) (See Supplementary Fig. S2b).

### Microscopy observations

To image the inside of *H. obtusa*, we used an optical microscope (BX51M, Olympus). To analyse the surface roughnesses of the bioinspired optical fibres, we used a laser microscope (LEXT OLS4000, Olympus).

### Measurement of light intensity

To evaluate the performance of the bioinspired optical fibres, we irradiated them in the dark and measured the illumination power transferred by them (See Supplementary Fig. S3). A solar simulator (XES-50S2-TT, Sanei Electric) was used as the light source. Light is captured by the scattering medium and is scattered and reflected and eventually guided to the optical fibre. The transferred light was detected by a photodiode (S2506-02, Hamamatsu Photonics). The detected light was converted into an electrical signal, which was passed through an operational amplifier. Finally, the resulting voltage, which was indicative of the light intensity, was measured with a data logger (X LR8431, HIOKI E.E.).

### Visualisation of light propagation in optical fibre

To verify the measured light intensity data, we visualised the process of light propagation through the optical fibre. A raw optical fibre and the optical fibre with the attached scattering medium were placed in a dark room and irradiated with a laser beam at incident angles, *θ*, of 0° and 90° using a laser (LP-130, PLUS) (See Supplementary Fig. S4).

## Supplementary information

Supplementary information
