# Supplementary material for: Lighting system bioinspired by Haworthia obtusa
Source: Sci Rep. 2020 Jul 9;10:11246. doi: 10.1038/s41598-020-68196-8 (PMC7347891; doi:10.1038/s41598-020-68196-8)
Supplement: Supplementary file 1 — Supplementary information [file 41598_2020_68196_MOESM1_ESM.docx]

**Lighting system bioinspired by *Haworthia obtusa***

Hiroki Gonome^1, *^, Kazuya Watanabe^2^, Kae Nakamura^3^, Takahiro Kono^2^, and Jun Yamada^2^

^1^Department of Mechanical Systems Engineering, Yamagata University, 4-3-16 Jonan, Yonezawa, Yamagata 992-8510, Japan

^2^Department of Mechanical Engineering, Shibaura Institute of Technology, 3-7-5 Toyosu, Koto-ku, Tokyo 135-8548, Japan

^3^Department of Precision Machinery Engineering, Nihon University, 7-24-1 Narashinodai, Funabashi, Chiba 274-8501, Japan

*Corresponding author

Tel.: +81-23-826-3103; E-mail: [gonome@yz.yamagata-u.ac.jp](mailto:gonome@yz.yamagata-u.ac.jp)

**Supplementary Informations**


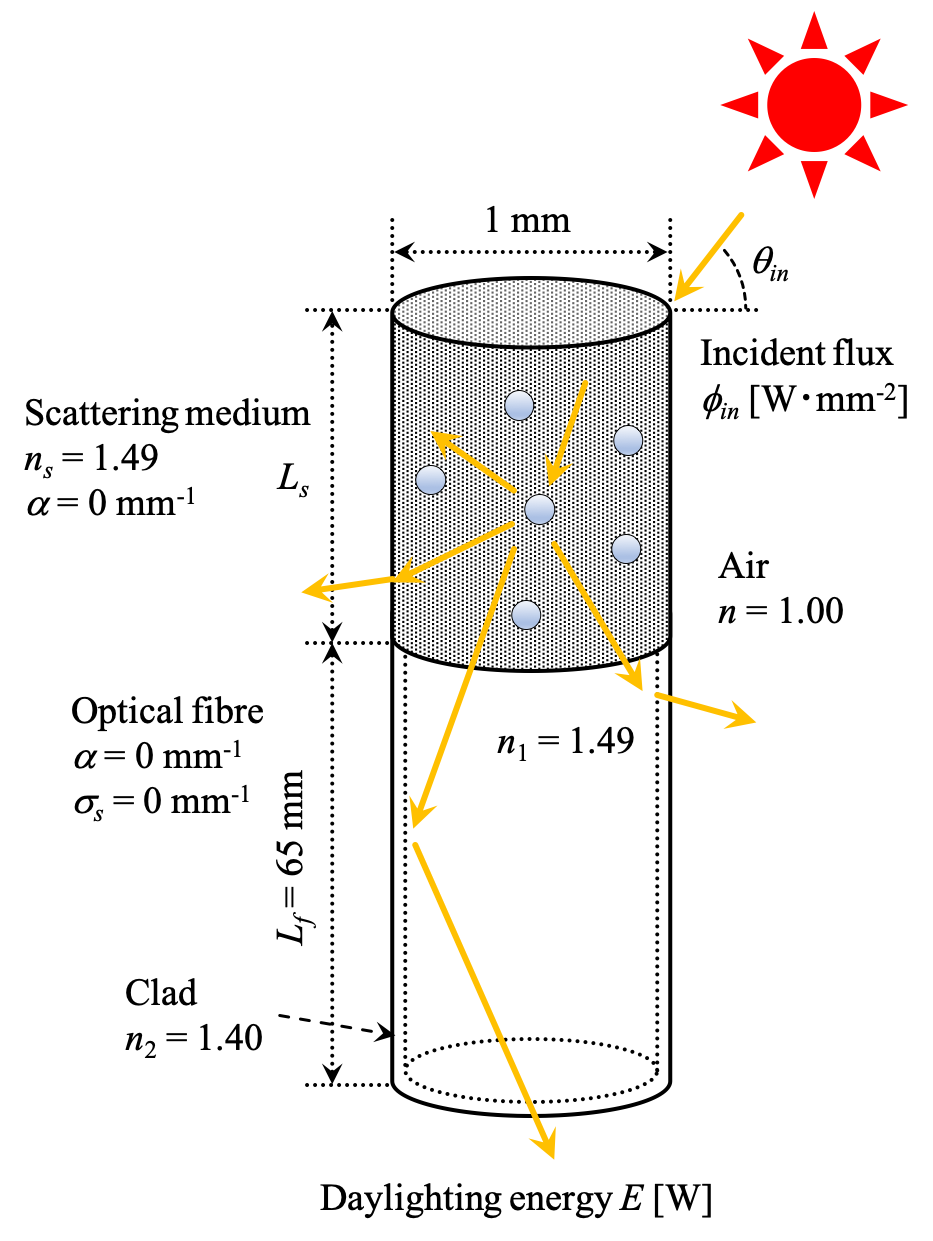


**Supplementary Figure 1 | Numerical model for radiative transfer in bioinspired optical fibre.** Diameter of optical fibre is 1 mm. Surrounding medium is air with *n* = 1.00. Refractive indexes of core, *n*_1_, and scattering medium, *n_s_*, are 1.49 while that of cladding, *n*_2_, is 1.40. Scattering coefficient of pellucid part is 0 mm^-1^. Light with incident flux, *φ*_in_, irradiates at angle *θ_in_*. Length of scattering part, *L_s_*, is variable. Scattering phase function is assumed to be isotropic, and scattering part is assumed to be nonabsorbing (*α* = 0 mm^-1^).


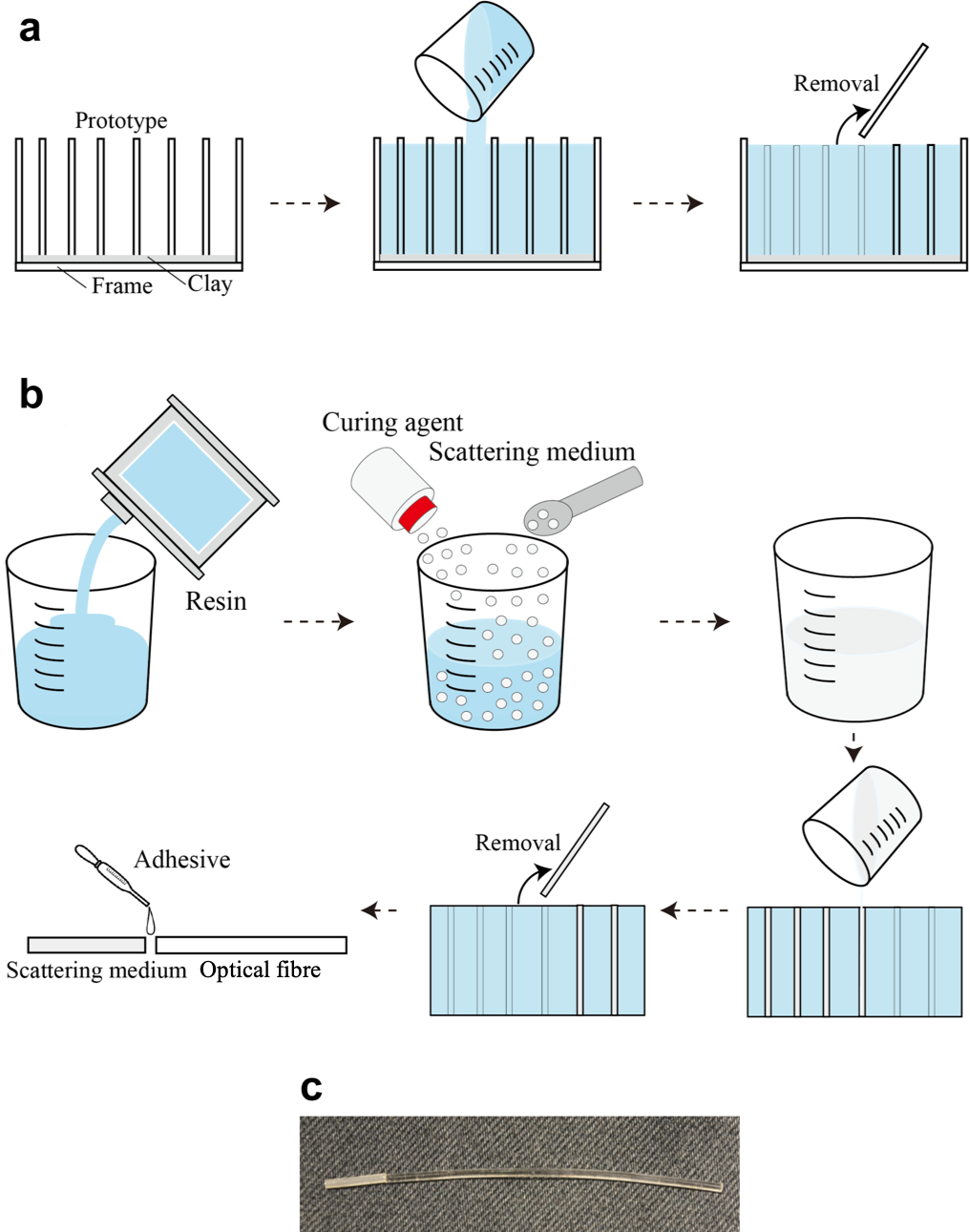


**Supplementary Figure 2 | Schematic of sample preparation.** (a) Fabrication of mould for scattering medium. (b) Fabrication of bioinspired optical fibre. (c) Photograph of bioinspired optical fibre.


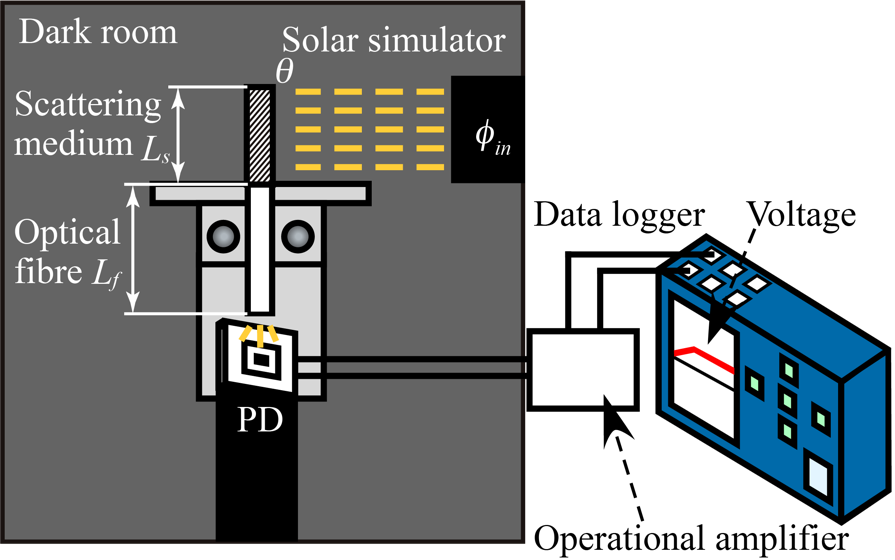


**Supplementary Figure 3 | Schematic of measurement of illumination power.**


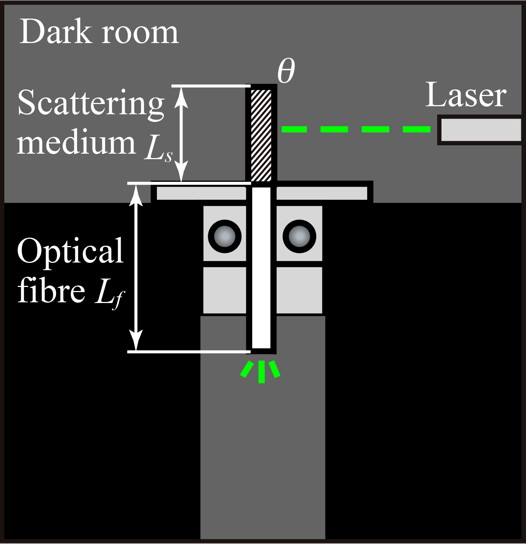

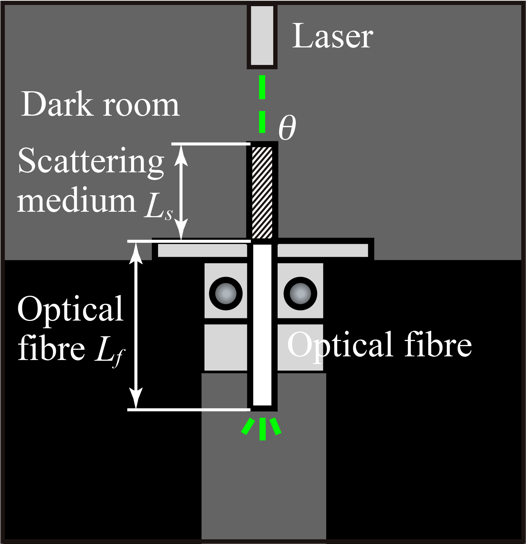


(a) *θ_in_* = 0˚ (b) *θ_in_* = 90˚

**Supplementary Figure 4 | Schematic of visualisation of light propagation in optical fibre.**
